# Supplementary material for: A Critical View of the Application of the APEX Software (Aqueous Photochemistry of Environmentally-Occurring Xenobiotics) to Predict Photoreaction Kinetics in Surface Freshwaters
Source: Molecules. 2019 Dec 18;25(1):9. doi: 10.3390/molecules25010009 (PMC7017383; doi:10.3390/molecules25010009)
Supplement: Supplementary file 1 [file molecules-25-00009-s001.zip › 3.2.4_gcc-4.4.0.zip!/3.2.4_gcc-4.4.0/3.2.4_gcc-4.4.0/doc/octave/html/interpreter/PKG_005fADD-and-PKG_005fDEL-directives.html]

PKG\_ADD and PKG\_DEL directives - Untitled


Previous: The INDEX file,
Up: Creating Packages


---

#### 35.4.3 PKG\_ADD and PKG\_DEL directives

If the package contains files called `PKG_ADD` or `PKG_DEL`
the commands in these files will be executed when the package is
added or removed from the users path. In some situations such files
are a bit cumbersome to maintain, so the package manager supports
automatic creation of such files. If a source file in the package
contains a `PKG_ADD` or `PKG_DEL` directive they will be
added to either the `PKG_ADD` or `PKG_DEL` files.

In `m`-files a `PKG_ADD` directive looks like this

```
     ## PKG_ADD: some_octave_command
```

Such lines should be added before the `function` keyword.
In C++ files a `PKG_ADD` directive looks like this

```
     // PKG_ADD: some_octave_command
```

In both cases `some_octave_command` should be replaced by the
command that should be placed in the `PKG_ADD` file.
`PKG_DEL` directives work in the same way, except the `PKG_ADD`
keyword is replaced with `PKG_DEL` and the commands get added
to the `PKG_DEL` file.
